# Nature in Your Face – Disruptive Climate Change Communication and Eco-Visualization as Part of a Garden-Based Learning Approach Involving Primary School Children and Teachers in Co-creating the Future

**DOI:** 10.3389/fpsyg.2020.568068

**Published:** 2020-12-18

**Authors:** Erica Löfström, Christian A. Klöckner, Ine H. Nesvold

**Affiliations:** Department of Psychology, Citizens, Environment and Safety, Norwegian University of Science and Technology (NTNU), Trondheim, Norway

**Keywords:** eco-visualization, school children, workshop, co-creation, disruptive communication, co-design

## Abstract

The paper describes an innovative structured workshop methodology in garden-based-learning (GBL) called “Nature in Your Face” (NiYF) aimed at provoking a change in citizens behavior and engagement as a consequence of the emotional activation in response to disruptive artistic messages. The methodology challenges the assumption that the change needed to meet the carbon targets can be reached with incremental, non-invasive behavior engineering techniques such as nudging or gamification. Instead, it explores the potential of disruptive communication to push citizens out of their comfort zones and into creative modes of re-creating futures. This is done by confronting us with consequences of consumption by means of art and eco-visualizations. The aim being to achieve awareness, mental flexibility, and spurring debate. Thus, we invite them to react – and act upon these reactions by communicating their feelings or thoughts. This is used as an entrance point for broader and/or deeper engagement in a structured three-step methodology; (1) Framing: A disruptive element is introduced into the local environment. This triggers an emotional reaction, which then is taken up in a process of framing the problem and working with solutions. (2) Twisting: in a guided process, the initial energy from the emotional confrontation is twisted into a creative exploration of innovative solutions, from the perspective of the children. (3) Using: The last stage is using the developed solutions in the local social system (or refining them further). The methodology is being applied in cooperation with a primary school, and is iteratively adjusted and evaluated in a formative, action-research based approach scenario. NiYF is to stimulate societal engagement through deliberately confronting stakeholders with unexpected and potentially upsetting appearances of nature, climate effects, or carbon-neutral lifestyle scenarios. We have verified the practical usefulness and potential of the methodology and found that NiYF triggers societal engagement and transition, negotiating responsibilities and unlocking action potentials. We also found that eco-anxiety, denial, self-efficacy and cognitive dissonance form children’s understanding of plastic. The project marks a paradigm shift from creating knowledge to using knowledge to create action, and a focus on learning from evaluating and adapting the approach in the field.

## Introduction

Municipalities are the custodians of vast public resources that, when managed properly, are instrumental in the development of cities that are happy, healthy and regenerative and the societal impact of these resources might depend on the quality of participation of and interaction with stakeholders. To facilitate such participation processes, the project described in this paper delivers a new methodology in the Garden Based Learning (GBL) domain, referred to as “Nature in Your Face” (NiYF), which is specifically aimed at working together across stakeholder groups to create solutions delivering shared value. It is built on the assumption that reciprocity among citizens is key to capturing the hearts and imaginations of all those who are part of the solution. At the core of the methodology lies a shift in mindset: away from the prevailing emphasis on not disrupting lifestyles, increasing comfort and making change as easy as possible (e.g., as proposed in the nudging concept) toward disruption and active work for radical changes. We believe that such a reorientation not only elicits participation but may even multiply the resources available to get the work done together as it also stimulates group processes and a feeling of belonging to a social group working toward a common goal as indicated by research on social identity and environmental action (Fritsche et al., 2017). Hence, a methodology that helps us take a leap forward and mobilize communities and resources in solving pressing issues may not only make sense as a tool for a limited application in a project case, but results may become part of the knowledge base that contributes to new ways of coping with barriers toward regenerative cities and this way of decision making might “infect” further sections of society. It may also increase acceptability for new and perhaps challenging policies as they in the ideal case emerge from within society and are not enforced from small and powerful interest groups. Based on these considerations, we investigate NiYF as a potential way of turning the tables by supporting municipalities in involving citizens, start-ups, innovators, businesses and organizations to become drivers of this development by demanding policy changes rather than simply adhering to ready-made, top-down solutions. This paper reports on the results of a case study involving school children to test and further develop the methodology.

Though being new in many respects, the NiYF project is building on the theoretical work of researchers from several disciplines. The level of change aimed for by the NiYF methodology can be described in the terminology of a societal transformation. Feola (2015) describes in a review paper several concepts of societal transformations driven by global environmental change, where especially the concept of deliberate transformation (O’Brien, 2012) and regime shift (Folke et al., 2010) are relevant for our context. Sharma (2007) outlines that a deliberate transformation is “a psycho-social process involving the unleashing of human potential to commit, care, and effect change for a better life” (see also O’Brien, 2012), which is in line with what NiYF is conceptualizing. Folke et al. (2010) distinguish between active transformation, which is a lower scale reconceptualization of some elements of a societal system while keeping the resilience of the overall system, from forced transformation, which is an imposed transformation not deliberately triggered by the actors in the system. NiYF tries to link these two elements: Triggering a forced transformation by stimulating a society with “unwanted” impulses, whereas then channeling that process over to an active transformation carried by the actors within the system.

On the more psychological level, NiYF builds on elements of social influence and group processes: Cialdini (2003) identifies social norms, social learning and social comparison as key elements for adoption of new ideas, which makes the aspect of social interaction an important component of the NiYF methodology. This is further supported by Abrahamse and Steg (2013) who found that social influence processes should involve direct social interaction to be successful. Transferred back to policy making, this implies that participation and autonomy should be strengthened, and be sustained over time (Bomberg and McEwen, 2012).

A third theoretical pillar behind the NiYF concept can be found in social practice theory (Shove et al., 2012). This theoretical branch assumes that people’s behavioral practices are rooted in a complex interaction of physical structures, regulations, and attached meanings. For NiYF, this means that the approach should challenge all three components to successfully change practices. Kalkbrenner and Roosen (2016) found that community projects may foster new norms, which might then change the meaning component of the social practices. Building on all three theoretical pillars, NiYF provides a strategy for co-creating and maintaining sustainable practices with policymakers and stakeholders by challenging assumptions and meanings, structures, and regulations through a social process which unlocks creative potential and societal resources, and triggers a societal transformation, if successful.

## Materials and Methods

### Transforming in Which Direction?

What has been stated so far raises the question, what kind of societal transformation NiYF would aim for. In the urgent need to address global climate change, scholars in the field have increasingly acknowledged the necessity to challenge the mantra of constant economic growth and rather limiting consumption (Ahuvia, 2017). Often consumption of goods is assumed to contribute to people’s happiness and life satisfaction (Zhang et al., 2014). However, compared to other daily activities, shopping in general scores only in the middle in terms of being an enjoyable experience (Zuzanek and Zuzanek, 2014). The most enjoyable activities are active sports, socializing and sex (Csikszentimihalyi and Hunter, 2003; Zuzanek and Zuzanek, 2014; Ahuvia, 2017). Furthermore, experiences with nature have often been found to contribute to well-being (Russell et al., 2013). Interestingly, all of these activities do not *per se* involve consumption, and interpersonal relationships has been found to be central for individuals’ enjoyment of these activities (Ahuvia, 2015). The NiYF methodology as a social activity could therefore in itself contribute to substitute consumption based with social activity-based happiness.

### Confronting Humans With Nature?

As stated in the previous paragraph, nature experiences are usually perceived as contributing to people’s well-being and happiness. However, in NiYF we conceptualize nature (also) in a different function, namely to create attention by disrupting established ways of being or seeing. NiYF utilizes eco-visualizations to achieve awareness, flexibility and of spurring debate (Löfström, 2014; Löfström and Svanæs, 2017). The eco-visualizations used in this project are not to provide neutral feedback, but designed to wilfully put people out of their comfort-zone by presenting nature or consequences of our societal use of resources in ways that are somehow disturbing. By using emotional stimuli in communicating we increase the possibility of people reacting to these concepts. However, this reaction is direct and – at the same time – transient. This means that if we present provocative eco-visualizations to members of the community, we invite them to react – and act upon these reactions by communicating their feelings or thoughts. However, in opening up difficult and disturbing themes and problems, we need a plan for harvesting these reactions. Hence, even though illustrating pressing issues, such as climate change, may well be useful as isolated events or installations to boost reflection and debate (Miles, 2010), NiYF harvests these reactions and carries them further in a social process.

The methodology uses provocative eco-visualizations because provocation lead to emotions, and emotions are more likely to lead to direct engagement than just information (Klöckner, 2015). Furthermore, once these emotions have been translated into engagement, this can be used as an entrance point for broader and/or deeper engagement. However, the emotional response to eco-visualizations typically do not last very long due to the process of getting familiarized with it, which counteracts its effects (see Löfström, 2008). This means that time is of the essence, and thus, we need to make use of the momentum and spontaneous reactions that come from the emotional stimuli (Klöckner, 2015). Ideally, the invitation and methodology that allows for people to discuss and engage in solving the issues raised should be presented in direct connection to the visualization itself. We open up a window of opportunity and need to start the process of harvesting this engagement.

### Three Methodological Pillars

First, as the methodology is to support municipalities’ ambitions to solve pressing issues in the community, it is necessary to define the overarching visions and goals together with the municipalities in question. This is done by the main researcher in cooperation with key competences in the municipality organization(s). The eco-visualizations should be developed to actualize this vision or goal in a confrontative or challenging way.

One of the problems with addressing extensive challenges, such as the growing amount of plastic in the ocean, is that the problem is so multi-faceted and complex. It becomes too large and the willingness to engage is largely dependent on both the perceived meaningfulness of peoples’ own engagement – i.e., the perceived ability to contribute anything meaningful to the discussion – and the perceived potential influence of this contribution to the discussion (Fritsche et al., 2017). Thus, limiting the scope of the challenge thematically or contextually helps overcome the “empty canvas syndrome,” i.e., the fact that total freedom may indeed be demotivating (Rosso, 2014). Asking people to go create without specifying the potential level of influence and the expectations on their part of such creative processes, will most likely lead to insecurity and unwillingness to contribute. Therefore, the first of the three pillars, framing, has the main intent of limiting the problem into something manageable.

Once we have framed the problem area to make it manageable, it is time for the next pillar, “twisting.” Again, giving people limitations or pre-defined themes to work with does not limit imagination. On the contrary, it helps open up for imagining a possible world; a temporary space, within which one can explore any set of ideas or possibilities (Gray et al., 2010; Dunne and Raby, 2013). This twisting state builds on specific challenges or scenarios that are set in relation to the previously framed contexts or themes. The intent of twisting is to get the participants into a creative and visionary mode. This is done by presenting goals that are more challenging than usual or by presenting truly ambitious or near impossible challenges.

Both the “framing” and “twisting” pillar may vary with the type and role of the participants. In Kristiansund, we tested the methodology in two sets of vision workshops. First, we arranged two separate workshops with local businesses and municipality employees. Secondly, we arranged two workshops at a local school with children in 5th grade. For this workshop, school children were chosen both to test the methodology in different settings, but also to get a different perspective from the more established “grown up” perspective. Both sets of workshops were led by the researcher, with the assistance of municipality employees.

The third, and last, pillar is “Using.” Using in this context means that the knowledge, ideas and solutions that have been developed as part of the previous two phases are used or taken forward either into actual decisions or are somehow included in the process of achieving the set goals of the municipality. The key issue here is not necessarily to implement all solutions, but to acknowledge the importance of these ideas and discussions and consider and/or elaborate on them. Communicating this influence is necessary to motivate participation (Støa et al., 2014) and to demonstrate that this is done ensures the transition from a one-off reaction to the eco-visualization to continued willingness of participation and of contributing to achieving the set goals.

### Research Data and Method of Analysis

The vision workshops and the interviews were audio recorded, and the researchers used field notes while aiding the pupils during their creative group work sessions. This vast material was then analyzed using the six-step process as developed by Braun and Clarke (2006). First, we got to know the data material, which this was done by listening to the audio files, transcribing and reading the transcripts and also by reading the field notes and discussing amongst the researchers. This resulted in an extensive amount of qualitative data. Thereafter, we coded the audio and field note-materials using a data analysis program (nVivo 12). Key themes were identified and are herein reported as part of the findings. In this paper, we have used actual quotes from the vision workshops and the interviews to illustrate the findings.

## Results of a Case Study on Plastic Waste

**FIGURE 1:**
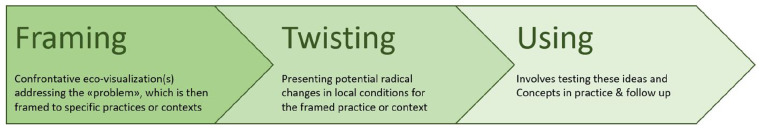
The three methodological pillars/phases.

In the case study, the researcher and problem owner (Kristiansund municipality) met at the Nordic Ideation Day arranged by Climate-KIC^1^. Here, Kristiansund municipality presented the problem of large amounts of plastic waste being brought ashore and the researcher suggested testing out the NiYF approach and methodology to support them in tackling this issue. The defined problem was then elaborated upon with regards to the potential use of the NiYF methodology. The discussions that followed led to an idea for a project that was presented to a jury by the researcher as part of this ideation day. The researcher managed to convince the jury of the project potential and won an award together with a promise of funding (15000 Euro) for a 3-month project. When the project goal was developed, the municipality already had an ambition of dealing with the large amount of plastic that floats ashore on their (long) shorelines. They also had local companies that were well underway to build a local plastic recycling plant. Even though it is still a major challenge to aim for plastic neutrality in Kristiansund, the large amount of plastic waste brought ashore by the Gulf stream and the fact that a local business is building a state-of-the-art plastic recycling plant was discussed in the project group. In this case, the confrontative eco-visualization was not difficult to achieve as nature is already doing it; the plastic waste is already very visible in the community and the contrast between Kristiansunds’ picturesque shoreline and the plastic is in itself already striking, We therefore showed pictures of huge piles of gathered local plastic waste to the workshop participants (see Figure 1) with examples of plastic waste art installations in the workshops. We also referred to previous headlines regarding this in the local press. For the case study, we did not create specific eco-visualizations and utilized the fact that plastic waste is in itself confrontative and alarming. The disruption was nevertheless successful and many of the children initially reacted with despair when they were told that the coastline pictures they were shown were actually from their own municipality. Many of the children were clearly troubled by what the future might bring:

I believe it will go under one day… because there is a lot of gasses that appear and it will be destroyed so that one can’t live on the planet anymore. (Vision workshop 2)

It destroys our future because we don’t know what will happen with plastic, and how the planet will become… I think it’s something with this plastic coming out and I’m a bit concerned for my future. (Vision workshop 2)

I’m afraid the world is going to end. No plants and that it sort-of becomes chaos. (Vision workshop 2)

Also, plastic was generally referred to as “natures vomit” and the planet was considered as being “sick” by the pupils. However, this description does not mean that the children perceive plastic as only a problem material as they later did problematize and understand also its advantageous properties (keeping food fresh etc.).

### Framing: “Nature’s Vomit”

**FIGURE 2:**
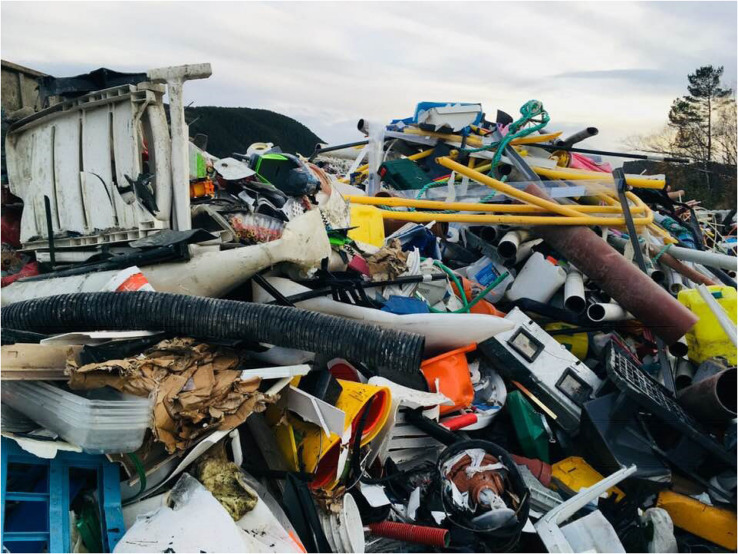
Photo from outside the local plastic recycle plant. In the background you can see the mountains. The plant is located just by the shoreline by an old industrial harbor (Photo: Erica Löfström).

The participants were recruited by the project groups’ municipality representatives via emails that were followed up by phone calls. One local primary school agreed to partake and invited us to hold the vision workshops as part of the school day. We carried out two different rounds of vision workshops with the schools’ 5th grade pupils. The first round was carried out between 12.00 and 14.00 h and over two adjacent days. The free minutes were carried out as on a normal school day. The original intention was to hold one workshop for each class on two different days, but after conferring with the teachers we decided to take on both classes during both days and instead divide the workshops in two parts. One reason for this was that the City Mayor could only partake in a prize ceremony for the children on day two and that the children would benefit from presenting their work for each other. Both class superintendents were present throughout both workshops and assisted in maintaining a normal school day structure. The children were presented with a contextual framing, which was co-developed with the teachers. This contextual framing was set to five different rooms or settings; at home, in the grocery store, in the classroom, in nature, and in the play context (toys). The 50 pupils were then divided into ten groups with five children in each (Kitzinger, 1994). As suggested by the teachers, we used the same work groups as had been used recently in another school assignment. This simplified the forming of groups as the children could relatively easily find their group members and sit down together. Each of the groups were later assigned with one room to focus on (see Figures 1 and 2). This meant that two groups of five were working on each of the five framed contexts. A second round of workshops was carried out the next school semester with the then new 5th grade pupils who were 36 in total and belonged to two different classes who were mixed in for the creative group work. This additional vision workshop had a similar structure as the previous one, but group interviews were added to also uncover which psychological mechanisms that underpins how children perceive the increasing amount of plastic littering in nature, when in the context of the vision workshops, i.e., as part of the NiYF methodology. Also, the second round of workshops only entailed one school day, due to practical issues. The presented results are from both rounds of vision workshops.

### Twisting: What If…?

**FIGURE 3:**
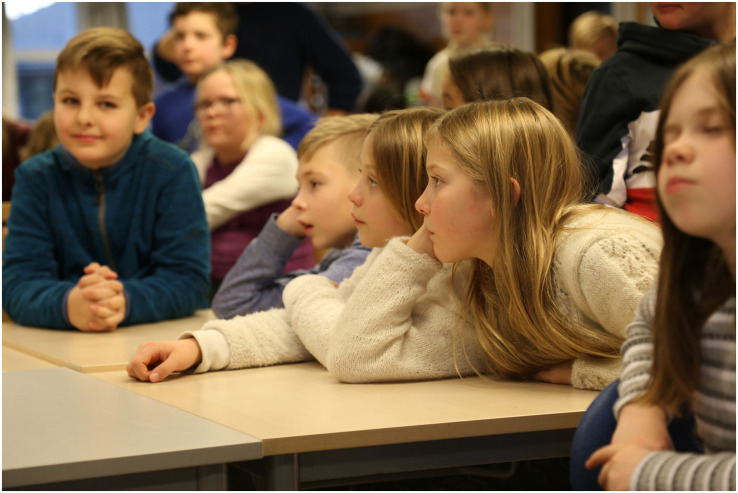
The children listening intently during the workshop introduction (Photo: Tore Lyngvær).

Once we had thematically framed the problem of “plastic waste,” the second methodological pillar, twisting, was introduced (see Figure 3). The children were instead encouraged to take on a pair of “plastic goggles” and to identify all plastic in their groups’ context or room. The children were not allowed to change rooms between the groups or to exchange group members as this was believed to risk taking focus away from the actual workshop theme. The twisting with regards to the children was to remove all plastic in their context. We also gave the example of replacing all plastic with something else and mentioned “wood” and “wool” as alternatives to consider. This challenge worked very well for all groups and helped boost imagination, which was demonstrated by the playfulness of the ideas that were later developed and presented by the children.

**FIGURE 4:**
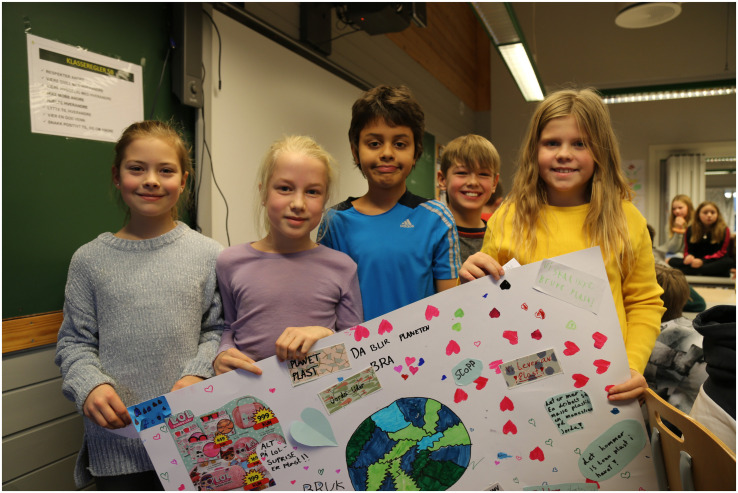
One of the groups holding their poster after sharing their ideas and results in the vision workshop (day 2) (Photo: Tore Lyngvær).

#### Using: Integration in Long-Term Strategic Work

The using pillar was only partly included in the case study due to the limited amount of time available (September 29–December 31, 2018, and February 5, 2020). As part of a full-scale project, and in the continuation of the Kristiansund case, the ideas and thoughts that were expressed in the workshops should be included in the municipalities’ continued work with the goal and vision and should also be continually followed up with communication both with the workshop participants directly, and as part communication and information to the general public, via social media as well as via other channels (including local press and media). Also, new workshops involving other and additional stakeholders in the community should be held as part of a long-term strategic work with the set vision and goals.

Amongst the children, each group was given the challenge of eliminating all plastic they could do without or replace in their specific “room” or context and were each given a large white paper (841 mm × 1,189 mm, A0) and had available a surplus of coloring pens, scissors, paper glue and scrapbooking materials, including magazines and promotion material from shops. The group works was initiated on the first workshop day and the children were given day two/the afternoon for finishing their assignment and present. All groups worked together for 90 min. The groups were facilitated by the core project team members and the teachers who rotated between the groups. On day two, for the first round of vision workshops, the children were also informed of the City Mayor’s visit and the forthcoming prize ceremony in a room that was used for school gatherings and performances. This motivated the children and clearly the prospect of winning some kind of award appealed to many of the groups. During the group works, all groups worked well and intensely with their task, and all produced posters that included both text and mixed-media illustrations. The children were more than willing to make do without many of the products that are part of everyday life, including video games and game consoles. To exemplify, one group that had the home as their room suggested to remove the resealable screwcaps on milk cartons. In the first workshop round, one group concluded – orally as well as in writing on their poster:

You grown-ups have made a mess – now it’s up to us children to make it right!

(The play/toy group A, Vision Workshop 1, day 2)

In the additional vision workshop, the qualitative interviews revealed that even though the children are aware of that previous generations did not have the knowledge we have today, they are nevertheless frustrated that *they* now will have to solve this problem:

In the old days, they thought it was fine to just throw away the garbage, but now it’s not okay. (quote from qualitative interview, Vision workshop 2)

It’s our ancestors that have done this, not us. But we will have to tidy up when we become adults (quote from qualitative interview, Vision workshop 2)

In the first round of workshops, both groups working within the classroom arena brought up the problem of not having recycling units for plastic and paper in their classroom. The school did, however, have access to recycling stations in the near vicinity and their suggestions were that they started recycling as soon as possible. The teachers confirmed that the classrooms would need containers for recycling different materials, but that the school budget did not include the cost of such a solution.

Many children also pointed out the fact that we did have plastic materials amongst the scrapbooking tools provided to them during the workshop, and we could only agree and ask for their advice in what materials to use instead. One of the “nature” groups suggested a special kind of “fishing” net:

We should have a fishing net that collects plastic but doesn’t harm the fish, and the fishermen could use these instead of ordinary nets. (quote from group work, vision workshop 1)

Of course, this idea cannot be put into practice without advanced technology development, but the idea demonstrates the willingness to experiment and to find solutions. Another group suggested they would build a one room beach hotel or cabin using only plastic waste from the ocean.

*We should build a cabin*^2^ *from the plastic. And then people could visit and see for themselves… (quote from group work, vision workshop 1)*

This cabin would illustrate the problem as well as being used to house tourists.

**FIGURE 5:**
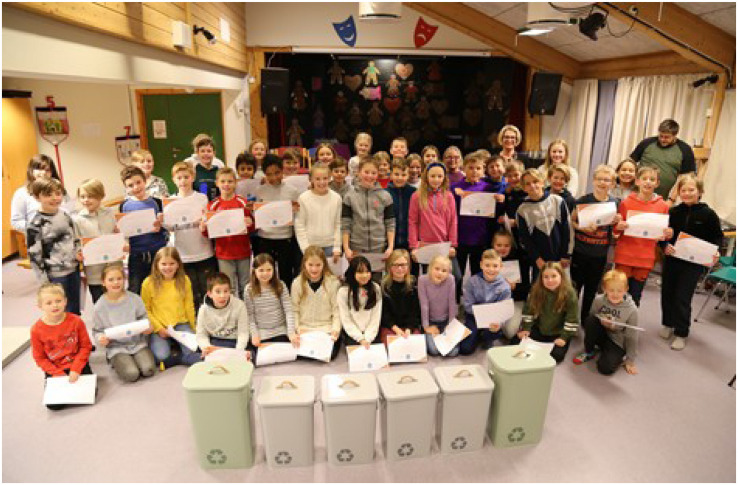
The children holding their individual diploma after getting positive feedback on their work from the researchers in plenum. The City Mayor, who handed out each diploma to the children, is present on the picture. In the foreground, the awards, waste recycling units for sorting plastic, paper and glass in the classrooms. The posters were mounted on the walls and left as exhibition (Photo: Tore Lyngvær).

After conferring with the teachers and the school headmaster, we addressed the lack of recycling bins for separating waste in the classroom by awarding the children with a waste sorting unit for each classroom. However, this was of course not a disruptive solution that came out of the project, but a way of rewarding the children for their participation in the study. The children were eager to get started with their recycling of plastic and other materials and the teachers had committed to support the recycling of the sorted materials. After conferring with the teachers, it was decided to declare all of the groups as winners, with a motivation for each group. After the prize ceremony all were served cinnamon buns and “julmust” in the auditorium (see Figure 5). During this time, four different children took the opportunity to ask if we would like to come back and visit them again.

### Psychological Mechanisms: How Children Perceive the Increasing Amount of Plastic Littering

In the second vision workshop, qualitative interviews were included as part of the group work, i.e., the researchers carried out interviews with the groups while they carried out the assignments. In hindsight, this “interruption” turned out to be a bit disturbing for the creative group works because the children really wanted to give correct and good answers to the questions. Possibly, the listening in and recording of quotes during the vision workshop group work (as was done in vision workshop 1) was a better way of gathering quotes during the vision workshops than the actual questions that were asked in group interviews for vision workshops 2. However, the interviews still helped uncover some psychological mechanisms that underpins how children perceive the increasing amount of plastic littering in nature, when in the context of the vision workshops, and ideally the interviews may perhaps be good to add as a separate step after carrying out the vision workshops. Nevertheless, the results of these interviews do show that there is a correlation between the findings and the theoretical framework, and that eco-anxiety, denial, self-efficacy and cognitive dissonance are all part of the children’s perception and understanding of plastic littering. Also, three prominent themes summarize how children perceive the amount of plastics used and plastic littering, namely (1) fear and frustration, (2) the amount of plastic and (3) that plastic is perceived as positive and negative, (i.e., the children recognize the advantages of plastic as material).

## Discussion

### A Critical Reflection on the Methodology

Since the Kristiansund case was the first application, we need to critically reflect, in how far the methodology with a confrontative step, followed by the framing, twisting, using steps contributed to solutions that other methodologies would not have provided. As already pointed out, the confrontative step was – mostly for reasons of time and budget restrictions – rather limited. Whereas the concept as outlined in the first section builds on unexpected confrontation with eco-visualization that gets people out of their comfort zone, the pilot project used visualizations created by nature itself. However, the labeling of these plastic piles as “natures vomit” framed them in a new way, which fueled the imagination of the workshop participants. In the following framing, twisting, using steps, this initial energy was taken on. Furthermore, it was very clear in our experience that the social aspect of the workshops both increased the perceived effect of the actions and the enjoyment and social support of the participants, empowering them. However, within the limited setup that could be realized in the pilot, the whole innovation potential of the methodology was not unlocked. The initial confrontation was rather limited, and since no artistic twist of the visuals was used, the potential for disrupting expectations and established assumptions was only partly taken out. The number of workshops and participants was limited and the transformative power of the process was thus limited as well. Most importantly, the continuity of carrying the transformation ideas further through the societal system needs to be established with stakeholders and the community in a more committing way as could be realized in the pilot.

## Next Steps: A Full-Scale Project

Based on the final reflections in the previous paragraph, the GBL NiYF methodology is perceived as promising, but needs more research and development to be fully effective. As the NiYF methodology is intended to be useful in addressing different challenges in different contexts, the methodology should be versatile and easily adapted to other challenges and various participant groups. To demonstrate the versatility of the methodology, and to give an indication of the road ahead, a case study as part of a full-scale research project has been drafted together with Trondheim Municipality (TK). TK has done extensive experimentation on participation, for instance through the 2017 Augmented Democracy Program^3^, which concluded that the complexity of the challenges make it difficult for citizens to fathom the options available to them. Hence, the challenge in Trondheim is to actively involve citizens in city development and planning with regards to a large number of specific challenges in the urban environment. The plan is to involve citizens, especially kindergartens^4^ in creating eco-visualizations on specific pre-defined themes located in hubs or stations along an urban pathway that runs through an area that is up for major (re-)developments^5^. Children will be provided with technical expertise, resources and support from relevant competencies (including local artists and authors) for realizing the eco-visualizations. The following table describes the hub concepts that will be used as a starting point for this second case study. The concepts have partly been developed by master-students of the Experts in Teams^6^ course on “alternative environmental communication^7^” and involve more radical disruptions of everyday life than could be implemented in Kristiansund.

The planned project in Trondheim uses the aforementioned three-pillar-methodology as part of a digital user interface for participation in city planning. Naturally, the methodology will be adjusted to work in a digital setting via an app for user involvement. The framing in this case will be thematic (see Table 1) while the twisting phase involves following up on these provoking and somewhat disturbing discussions via discussions and challenges. The using phase consists partly on actual changes and influence on the city development. In addition, the using is communicated via the digital interface and via social media.

**TABLE 1:** The preliminary eco-visualizations developed for hubs along the urban pathway.

**Eco-visualization concept**	**Description; supports discussions on**
Lung trees: visualizing sensor-based air pollution data	Data is visualized as lungs in trees that are breathing in and out. Visual movement is accompanied by sounds of breathing that becomes more strained as the air quality decreases. Supports discussions on how to improve the air quality by minimizing traffic.
Whispering forest: poetry and storytelling	Trees are whispering poetry and telling stories in a serene spot. Users can add own content. Supports discussions on the potential use of urban spaces.
Human zoo: human behavior under the magnifying glass	Watching humans as they hurry to and from buses and pass by in their cars on one of the busy traffic spots of Trondheim from inside a “green pocket.” The contrast of the serene nature in a green pocket and the busy life outside is illustrated by a window in the green wall. Supports discussions on means of transport and traffic solutions.
Nature strikes back: old buildings	An old building in a bad condition is located along the path. By means of light projection, it will appear as if it falls down, brick by brick, nature reclaims it. Supports discussions on the future of poorly maintained buildings.
Nature strikes back: reclaimed infrastructure	The old Sluppen car bridge will be remade for pedestrians and cyclists as part of the new Sluppen area. Sensors trigger a partial “flooding” of the bridge, and microorganisms or fish will jump over the passing cyclist. Supports the discussions on loss of comfort or convenience and switches the scales.
Big city data light-show	The newly built Sluppen Lysgård (http://www.lysgarden.no/?gclid=EAIaIQobChMIiLnmi8PB2QIVBkMZCh0-QAyEEAAYASAAEgJ7XfD_BwE) has advanced light projection technology that is put to our disposal by our cooperation partner Kjeldsberg. This enables us to visualize big data (traffic et cetera) in creative ways. Supports discussions on a holistic city scale.

The full-scale project has been granted funding from the Norwegian Research Council (NFR) and will start up in September 2020 (project nr 302111). In addition to the herein described case study on mobility, the full-scale project involves three more cases (housing, food, and plastic). The NiYF methodology will be iteratively adjusted and evaluated in a formative, action-research based approach. The CO2 savings of each realized case will be assessed by LCA based environmental scenario analyses and an upscaling assessment will be conducted.

## Conclusion

In the project described herein, we have tested and further developed the NiYF approach in relation to a specific pressing environmental issue and challenge, in a Norwegian municipality. By realizing the NiYF idea in the Kristiansund case, and demonstrating its feasibility, we have verified the practical usefulness and potential of the methodology, but also identified critical issues for its implementation. We feel confident that the NiYF methodology is versatile enough to be successfully used in other (municipality) contexts and in addressing many of the worlds pressing environmental issues. The project goal that is to be supported by the NiYF methodology needs to be sufficiently founded in the problem owners’ organization, as the experiences from Kristiansund show where the results are only partly taken further. Therefore, starting with an actual problem that is already at least partially defined in the municipality is an advantage. Preferably the problem or issue that is to be solved should be a major priority in the municipality. If not, it may be difficult for the municipality to put work hours and resources into the project and it may take a long time to approach dealing with a specific challenge and set ambitious goals to achieve. If the defined problem or issue is already visible in the community, as it was in Kristiansund, this is of course an advantage with regards to confronting people with the problem. However, creating eco-visualization concepts to illustrate less obvious topics or to highlight already present problems is indeed a possibility, and it might be an arena where the NiYF approach unfolds its true potential. There is a growing number of artists addressing environmental issues and climate change in different ways^8^. The use of technology to visualize sensor data such as air pollution or traffic patterns is but one possibility. As mentioned earlier, the Kristiansund case-study will be used as a starting point for additional projects. Based on the results of the performed case study, the NiYF methodology may offer a way forward that does acknowledge the need for major changes in at individual and societal level and invites citizens, municipalities, businesses and research to co-create visions and solutions finding a needed new pathway that shifts the focus away from the prevailing emphasis on retaining comfort toward an emphasis on engaging in new, maybe disruptive solutions. In particular, the children proved to be more than willing to limit consumption, and the happiness, innovativeness and creativity that was displayed during all vision workshops far exceeded the researchers’ expectations, also underlining the important social aspect of the methodology for creating a momentum. Using this experience as a point of departure, we are confident that the NiYF methodology successfully approaches the need for major changes in the way we use resources on individual and societal level and offers a way forward. It confronts individuals and groups with the challenges of global climate change by means of confrontative eco-visualizations, stimulates their social interactions – which in itself is a contribution to non-consumption based well-being – and offers a stepwise path forward to achieve a joint mobilization of resources and ideas in solving the challenges we are facing. Hopefully, the results of future projects will provide not only a methodology that contributes to new ways of coping with the barriers of creating regenerative cities but also deepen the understanding on how to codesign and gain acceptability for new and perhaps challenging policies.

In the scientific domain, the project marks a paradigm shift from creating knowledge to using knowledge in order to create action, and a focus on learning from evaluating and adapting the approach while being in the field. This will also provide important new knowledge for the scientific community about the potential of the innovative concept of disruptive communication. NiYF is to stimulate societal engagement through deliberately confronting citizens and stakeholders with unexpected and potentially upsetting appearances of nature, climate effects, or carbon-neutral lifestyle scenarios in an artistic way. This will be followed up with a structured process of co-creating action capacity in local communities through inclusive vision workshops (in which future lifestyles will be explored and negotiated), thus removing barriers and changing attitudes and behaviors as specified in the call. NiYF triggers societal engagement and transition, incorporating political values, negotiating responsibilities and unlocking action potentials, thus addressing the need for studies that include diverse interests and stimulate sharing responsibilities.

## Data Availability Statement

The raw data supporting the conclusions of this article will be made available by the authors, without undue reservation.

## Ethics Statement

The studies involving human participants were reviewed and approved by NSD approval (Norwegian national ethics review). Written informed consent to participate in this study was provided by the participants’ legal guardian/next of kin. Written informed consent was obtained from the minor(s)’ legal guardian/next of kin for the publication of any potentially identifiable images or data included in this article.

## Author Contributions

EL wrote the manuscript with valuable contributions from CK and IN. All authors contributed to the article and approved the submitted version.

## Conflict of Interest

The authors declare that the research was conducted in the absence of any commercial or financial relationships that could be construed as a potential conflict of interest.

## Footnotes

1 https://www.climate-kic.org/events/nordic-ideation-day/

2 Hytte, norwegian word for holiday home.

3 https://www.facebook.com/688648314498316/posts/1970517426558838

4 https://www.trondheim.kommune.no/oya-bhg/

5 https://www.trondheim.kommune.no/aktuelt/kunngjoring-arealplan/andre-planer/kommunedalplan-for-sluppen-k20180033/

6 https://innsida.ntnu.no/wiki/-/wiki/English/experts+in+teamwork

7 https://www.ntnu.no/eit/psy3809

8 https://www.nytimes.com/2018/08/22/t-magazine/climate-change-art.html
